# Neurosarcoidosis Presenting With Confusion and Speech Alteration

**DOI:** 10.7759/cureus.42627

**Published:** 2023-07-28

**Authors:** Sara Heard, Flavia Miller Machado, Jai Juganya Ponnusamy

**Affiliations:** 1 Internal Medicine, MetroWest Medical Center/Tufts School of Medicine, Framingham, USA; 2 Neurology, MetroWest Medical Center/Tufts School of Medicine, Framingham, USA

**Keywords:** facial palsy, speech alteration, confusion, high-dose steroids, aseptic meningitis, leptomeningeal enhancement, neurosarcoidosis

## Abstract

Neurosarcoidosis (NS) is a rare manifestation of sarcoidosis, a multisystem inflammatory granulomatous disease. We describe a unique case of NS with confusion and speech alteration as presenting symptoms. A 65-year-old male with a history of Ramsay Hunt syndrome and Lyme infection presented to the emergency room after an acute episode of disorientation, garbled speech, and left facial droop, along with months of worsening generalized fatigue, gait ataxia, left-sided periorbital headaches, bilateral peripheral neuropathy, and bladder disturbance. A recent CT scan of his chest showed mediastinal lymphadenopathy, and a lymph node biopsy revealed non-necrotizing granulomas, Langhans giant cells, and focal Schaumann bodies. A brain MRI revealed a mildly enlarged anterior pituitary gland, mild prominent enhancement of the trigeminal nerves bilaterally, and right frontal, parietal, and superior temporal leptomeningeal enhancement. Lumbar puncture cerebrospinal fluid analyses were consistent with aseptic meningitis. A diagnosis of probable NS was made. The patient received IV methylprednisolone 1 g for three days, followed by a prednisone taper with clinical improvement. NS is a diagnostic challenge due to the variability of clinical presentations of the disease. This case demonstrates how vague chronic neurologic symptoms preceding an unusual acute clinical presentation delayed the diagnosis of NS in a patient with sarcoidosis.

## Introduction

Neurosarcoidosis (NS) is a rare manifestation of sarcoidosis, a multisystem inflammatory granulomatous disease. Neurologic complications arise in roughly 5-10% of patients with sarcoidosis [1,2]. Common clinical presentations include cranial neuropathy, encephalopathy, peripheral neuropathy, myopathy, neuroendocrine dysfunction, myelopathy, radiculopathy, focal/generalized seizures, and aseptic meningitis. NS remains a diagnosis of exclusion and is based on the clinical profile supported by pathological evidence of non-caseating granulomas since there is no high-accuracy biomarker available [3]. NS is typically a monophasic illness; however, some patients have a relapsing-remitting course [2]. The clinical manifestations of NS are variable, which oftentimes creates a diagnostic conundrum. Additional case reports are needed to better elucidate the variable presentations of the disease to enable prompt diagnosis and treatment.

## Case presentation

A 65-year-old male with a history of Ramsay Hunt syndrome and Lyme infection presented to the emergency room after an episode of altered speech and confusion. The patient’s wife found the patient unresponsive at breakfast, holding his head in his hands. After several seconds when he responded, his speech was slurred, garbled, and incomprehensible with left facial droop and drooling from the left corner of his mouth. His left-sided facial droop and altered speech resolved by the time he arrived at the hospital. The NIH stroke scale was 0 during the initial neurology evaluation, and, therefore, there was no indication for IV tissue plasminogen activator (tPA). CTA head/neck was negative for large vessel occlusion, and CT brain showed no acute intracranial process but did reveal pituitary enlargement.

The patient endorsed worsening generalized fatigue over the past six months necessitating him to leave his technology job on disability. He underwent several evaluations by his primary care physician for frequent falls, gait imbalance, and one episode of loss of consciousness associated with orthostatic hypotension. He also reported worsening intermittent left-sided periorbital headaches, with occasional photophobia and nausea, as well as new onset bilateral foot and hand paresthesias, for which he had an electromyography nerve conduction study revealing mild peripheral neuropathy. He endorsed increased urinary frequency, worsening over the last several months. Three months prior, a CT scan of his chest with his pulmonologist for workup of dyspnea and malaise showed mediastinal lymphadenopathy and subsequent lymph node biopsy revealed non-necrotizing granulomas, Langhans giant cells, and focal Schaumann bodies, non-specific but compatible with a diagnosis of sarcoidosis.

**Figure 1:**
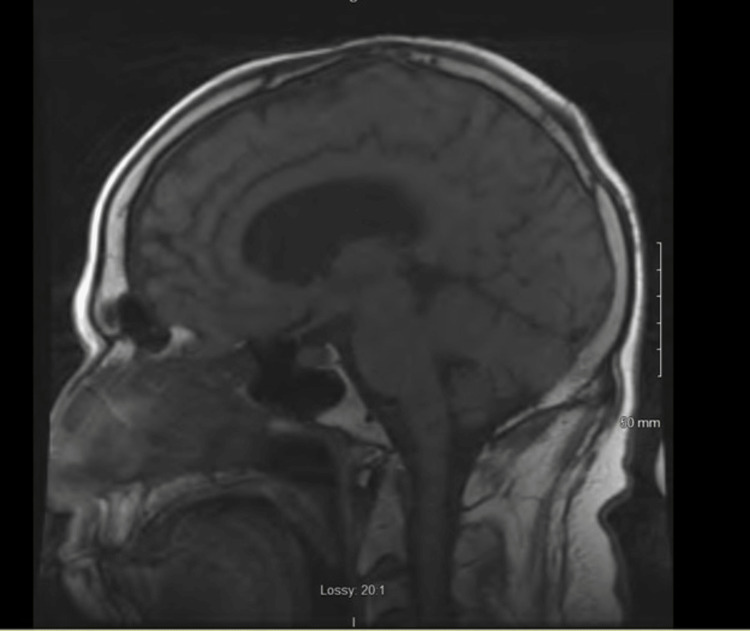
Sagittal MRI showing mild anterior pituitary enlargement, suggestive of possible anterior hypophysitis

**Figure 2:**
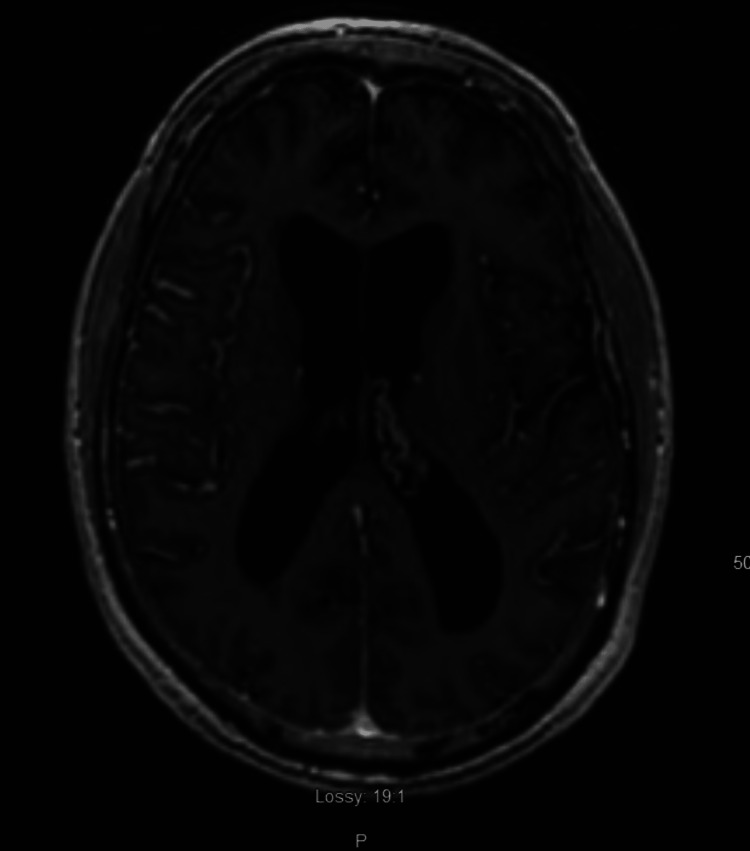
Axial MRI images demonstrating leptomeningeal enhancement in the insular cortex and frontal cortex

**Figure 3:**
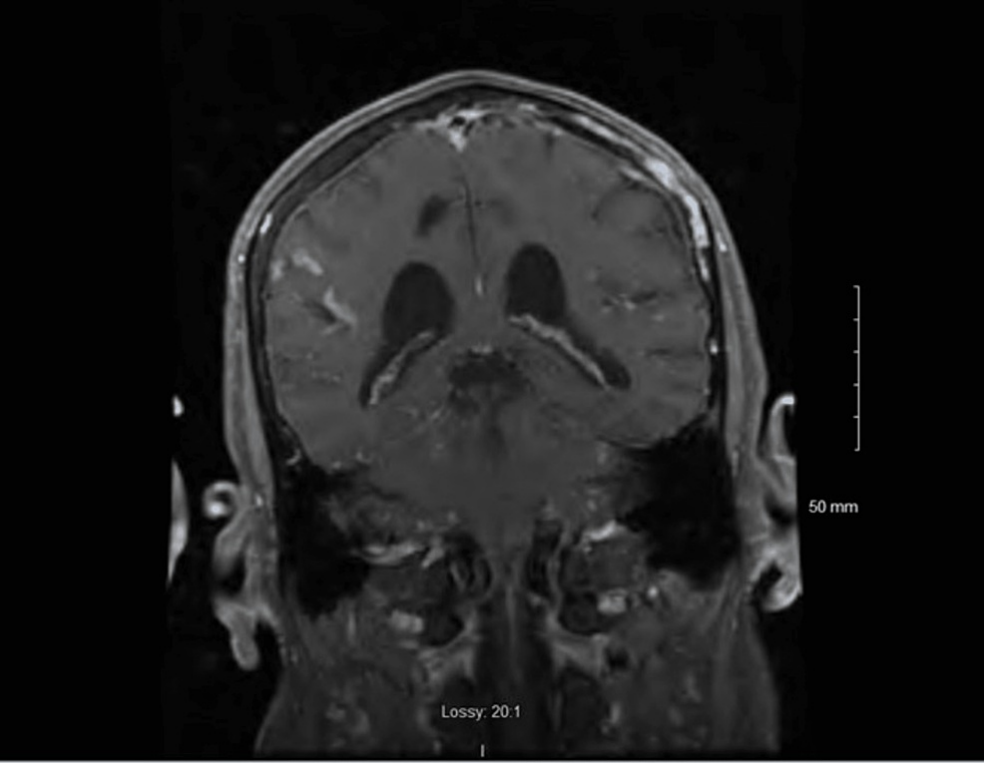
Coronal MRI showing temporal leptomeningeal enhancement

On further investigation, a brain MRI revealed a mildly enlarged anterior pituitary gland (Figure 1), mild prominent enhancement of the trigeminal nerves bilaterally, and right frontal, parietal, and superior temporal leptomeningeal enhancement (Figures 2-3), suggestive of probable NS. The MRI was not dedicated to the pituitary gland and, therefore, was done without gadolinium on the sagittal cut. A lumbar puncture was performed to exclude any other etiologies of leptomeningeal enhancement. Cerebrospinal fluid studies demonstrated mildly elevated white blood cells (13 cells/uL), mildly elevated glucose (82 mg/dL), mildly elevated total protein (82 mg/dL), and no oligoclonal bands. CSF cytology showed reactive lymphocytic proliferation. Electroencephalogram revealed frequent episodes of diffuse theta and delta slowing, consistent with diffuse cerebral disturbance as seen in an encephalopathy of any etiology with no epileptiform or lateralizing abnormalities. The erythrocyte sedimentation rate was elevated (32 mm/hr) and CRP was normal (4.0 mg/L). Treponema pallidum antibody was negative, and HIV 1 and 2 antigen and antibody were negative.

The differential diagnosis of an acute episode of altered mental status and incomprehensible speech is quite broad but most notably includes transient ischemic attack, complex partial seizure, acute viral meningoencephalitis, and NS. Initial head CT ruled out acute intracranial hemorrhage or large territory infarct, and resolution of symptoms upon arrival to the ED with no focal neurologic deficits eliminated the indication for IV tPA. The patient was started on aspirin 81 mg daily and levetiracetam 500 mg BID because a transient ischemic attack and complex partial seizure could not be ruled out given the acute onset of facial droop, altered speech, confusion, and memory loss, as well as the increased risk of focal seizure in the setting of the leptomeningeal enhancement structural MRI findings. However, the patient had no tongue laceration, or bowel/bladder incontinence, and the EEG showed no evidence of epileptiform discharges. However, the presence of NS lesions in the central nervous system, especially the brain parenchyma, could be foci for seizure activity; therefore, the patient could have had a complex partial seizure in the setting of NS, which wasn’t captured on EEG. Although neurovascular complications of sarcoidosis are rare, it is also possible that the patient could have had a transient ischemic attack in the setting of NS. The lack of meningeal signs, fever, or elevated WBCs decreased the likelihood of acute viral meningoencephalitis, which was confirmed via lumbar puncture. CSF studies were consistent with aseptic meningitis, which is typical of NS. HIV and anti-treponemal serologies were negative which ruled out HIV-associated neurocognitive changes or neurosyphilis.

The patient was treated for presumed NS with IV Solumedrol 1 g for three days. He tolerated the steroids with no adverse effects, and his headache improved throughout the hospital stay. He was also started on levetiracetam 500 mg twice daily due to strong suspicion that the episode that brought the patient into the hospital was a focal seizure with loss of awareness. On discharge, he was prescribed a prednisone taper starting with 60 mg PO daily and Bactrim DS daily for *Pneumocystis jirovecii *pneumonia prophylaxis in the setting of likely chronic steroid use. It was recommended that he follow up with neuroimmunology and pulmonology in the outpatient setting. Three weeks later, the patient reported near complete resolution of his neurologic symptoms.

## Discussion

A presumed diagnosis of NS was made based on the patient’s acute episode of confusion, speech alteration, left facial nerve palsy with preceding six months of worsening headaches, peripheral neuropathy, gait ataxia, and polyuria after a recent mediastinal lymph node biopsy confirming non-necrotizing granulomas. Leptomeningeal enhancement, pituitary enlargement, and trigeminal nerve enhancement on brain MRI, as well as CSF analyses demonstrating aseptic meningitis, further validated our diagnostic suspicion. Clinical improvement after treatment with glucocorticoids reinforced our previous diagnosis of NS.

This patient had a history of Ramsay Hunt syndrome several years ago and Lyme disease the year before this hospitalization. Both herpes zoster oticus and Lyme disease can cause unilateral facial paralysis. There was a clinical question as to whether the current acute episode of left-sided facial droop was in some way related to his past viral infection and tick-borne illness. The patient reported that his unilateral facial paralysis and ear pain due to the reactivation of latent varicella zoster virus resolved after antiviral treatment. He had a classic bull’s eye rash and was treated with antibiotics for Lyme infection but did not develop a unilateral facial palsy at that time. There is a single reported case of bilateral facial nerve palsy that was initially thought to be Ramsay Hunt syndrome and the patient was started on acyclovir, then was thought to be possibly acute invasive fungal rhinosinusitis but was eventually diagnosed as NS [4]. However, there have yet to be published cases demonstrating that previous Ramsay Hunt syndrome or Lyme disease could be related to the development or increased susceptibility for facial nerve palsy as a neurologic manifestation of sarcoidosis.

Acute onset of confusion has been reported as a presenting symptom of NS of the central nervous system [5]. However, our patient’s chronic vague symptoms involving both the central and peripheral nervous system followed by the acute onset of confusion and altered speech makes our clinical case unique. The incomprehensible speech could have been due to altered mental status, transient aphasia due to central nervous system involvement, or dysarthria in the setting of facial neuropathy (peripheral nervous system involvement). Facial neuropathy is the most common presenting symptom and accounts for 70% of cranial neuropathies in cases of sarcoidosis [6]. He also had radiographic evidence of trigeminal nerve inflammation (enhancement of the trigeminal nerves bilaterally), another form of cranial neuropathy, but was asymptomatic since he did not have facial pain. He had headaches and cognitive changes, which could be attributed to meningeal disease and/or parenchymal involvement. He also had multiple preceding months of orthostatic hypotension/ dysautonomia, bilateral hand, and foot paresthesias, as well as gait imbalance, which are features of small and large fiber neuropathies. It is also quite possible that the patient’s increased urinary frequency could be related to NS-associated neuroendocrine dysfunction in the setting of hypophysitis. Additional workup will be needed to investigate this further.

Our patient’s central and peripheral neurologic symptoms improved with three days of stress dose steroids, followed by a prednisone taper, and, therefore, didn’t require immunosuppressant or biologic therapeutic alternatives. The prognosis is usually good in cases of facial nerve palsy, and complete recovery has been reported in 90% of patients [1]. The first-line treatment option for NS is glucocorticoids and the dosing and duration depends on the symptomatology. CNS involvement requires high-dose glucocorticoids 1 mg/kg/day, whereas peripheral and cranial neuropathies may only require moderate-dose glucocorticoids 0.5 mg/kg/day. Rapidly deteriorating patients with paralysis, visual loss, or altered mental status may require stress-dose IV methylprednisolone 1 g per day for three to five days [1]. However, there is a chance of recurrence of NS symptoms once the steroids are tapered [7]. In these patients, conventional synthetic disease-modifying antirheumatic drugs are considered second-line treatment, and anti-tumor necrosis factor (anti-TNF)/cytotoxic agents are third-line options. Extreme cases have benefitted from cranial irradiation [8], and mortality due to NS has decreased with the advancement in biologics.

## Conclusions

Diagnostic uncertainty is an ongoing challenge for clinicians encountering patients with NS due to the wide variability in clinical presentation. Although the differential diagnosis was broad for our patient, the constellation of clinical symptoms, imaging results, and laboratory/histopathological findings made a final diagnosis of NS most probable. This case demonstrates how a patient with initial subacute nonspecific neurologic symptoms can delay diagnosis of NS; however, once more acute symptoms develop in the progression of the disease, the constellation of symptoms can be more recognizable, allowing for successful treatment of NS. Additional NS case reports illustrating the variability in clinical presentation are needed for faster diagnosis, deepening of our understanding of the disease process, and advancement in treatment options to improve patient outcomes.
